# Study on measurement method for apple root morphological parameters based on *Labview*

**DOI:** 10.1186/s13007-019-0535-4

**Published:** 2019-12-11

**Authors:** Yu Liu, Ji Qian, Xin Yang, Bao Di, Juan Zhou

**Affiliations:** 1 College of Horticulture, Hebei Agricultural University, Baoding, Hebei China; 2 College of Resources & Environment Sciences, Hebei Agricultural University, Baoding, Hebei China

**Keywords:** *Labview*, Apple root system, Morphological parameters, Image preprocessing

## Abstract

**Background:**

Traditional measurements of apple seedling roots often rely on manual measurements and existing root scanners on the market. Manual measurement requires a lot of labor and time, and subjective reasons may cause the uncertainty of data; root scanners have limited scanning size and expensive. In case of fruit roots, coverage and occlusion issues will occur, resulting in inaccurate results, but our research solved this problem.

**Results:**

The background plate was selected according to the color of the seedling roots; the image of the roots of the collected apple seedlings was preprocessed with *Vision Development Module* by combining image and *Labview*. The root surface area, average root diameter, root length and root volume of apple seedlings were measured by combining root characteristic parameters algorithm. In order to verify the effectiveness of the proposed method, a set of measurement system for root morphology of apple seedlings was designed, and the measurement result was compared with the Canadian root system *WinRHIZO 2016 *(Canada). With application of *SPSS v22.0* analysis, the significance *P* > 0.01 indicated that the difference was not significant. The relative error of surface area was less than 0.5%. The relative error of the average diameter and length of the root system was less than 0.1%, and the relative error of the root volume was less than 0.2%.

**Conclusions:**

It not only proved that the root surface area, average root diameter, root length and root volume of apple seedlings could be accurately measured by the method described herein, which was handy in operation, but also reduced the cost by 80–90% compared with the conventional scanner.

## Introduction

Apple is one of the four major fruits in the world. Its root system architecture constitutes an important part of apple seedlings, which is buried deep in the soil to fix plants as the main vegetative organ for plant growth and metabolism. The morphological parameters of root mainly include the morphological parameters of main root, lateral root and fibrous root of the seedling. The root of the diaphysis is the main root, and the branches on it are collectively referred to as lateral roots. The fine roots formed by several branches on the lateral roots are called fibrous roots. As the main factors reflecting the growth of roots, they have an impact on shoot and leaf growth aboveground, carbon assimilation, flower bud differentiation through absorption of water, nutrients and endogenous hormones. Neri et al. showed that lateral roots could be formed anywhere along the primary roots of strawberry plants from a few stem cells distributed along the pericycle close to the protoxylem arches [1]. The tree body also establishes a close connection between matter and energy exchange through roots and soil during its life activities. The structure and form of root system is affected by the physical and chemical properties of the soil, the soil microbial factors of soil and other parts of the tree body. According to the research made by Polverigiani et al., soil sickness was a widespread problem in replanted apple orchards with a complex symptomatology and etiology influenced by soil and climate conditions. Their research also showed that there was a significant interaction between soil treatment and sampling site and root growth was correlated with the organic matter content in the soils [2]. Moreover, the root is buried deeply in the ground, so the features perennation, complex structure and huge form, which makes it harder for people to research on apple roots in-depth, systematic and comprehensive than on the aboveground part. Periodical measurement and analysis of the root morphology parameters of plants have great significance for understanding and studying the growth and development of plants, as well as controlling pests and diseases.

Traditional measurements of apple seedling roots often rely on manual measurements and existing root scanners on the market. Manual measurement requires a lot of labor and time, and subjective reasons may cause the uncertainty of data; root scanners have limited scanning size and expensive. In case of fruit roots, coverage and occlusion issues will occur, resulting in inaccurate results, but our research will solve this problem.

Machine vision technology is a comprehensive technology involving many disciplines such as artificial intelligence, intelligent control, neural network, neurobiology and graphic image processing. With the development of machine vision technology and research in the field of plant science, it has been widely used in the identification of plant species, plant growth information detection, quality inspection and classification of agricultural products as well as visual navigation of farmland. For example, Sunoj et al. proposed measuring the size of sunflowers by machine vision in 2018 [3], Qinghua et al. put forward to classify potato quality by machine vision in 2018 [4], Gongal et al. proposed estimating the size of apple fruit by 3D machine vision in 2018 [5], Benoit et al. simulated image acquisition in machine vision for seedling elongation, root segmentation algorithm for image processing validation in 2014 [6]. The application of machine vision-assisted electronic image analysis systems increases the precision and efficiency of plant production and avoids the subjective effects of human observation. This research combined the using of *Labview* and the preprocessing of the image taken by camera acquisition assisted with machine vision technology. The root morphology parameters-surface area, length and volume of apple root were measured by the designed system. It provided important data for the researchers to get the growth status of apple seedling roots, as well as the physical and chemical properties of soil in order to improve the cultivation and management of apple seedlings.

## Results

### T Test

The "Independent Sample T-Test" process test is primarily used to test whether two samples are from a subject with the same average. In this study, it was mainly used to detect whether the average value between the *Labview* calculated value and the two *WinRHIZO 2016* groupings was the same.

In this study, independent sample T-tests were carried on root surface area, average root diameter, root length, and root volume by *IBM SPSS v22.0* (see Table 1).

**Table 1:** Independent sample test

	Levin test		T-test for mean equality						
	*F*	Significance	*t*	Degrees of freedom	Significance (double tail)	Average deviation	Standard error difference	99% confidence interval of the difference	
								Lower limit	Upper limit
Surface area	0.020	0.889	0.073	58.000	0.942	2.779	38.061	− 98.589	104.146
			0.073	57.992	0.942	2.779	38.061	− 98.590	104.147
Diameter	0.003	0.955	0.013	58.000	0.990	0.001	0.080	− 0.211	0.213
			0.013	58.000	0.990	0.001	0.080	− 0.211	0.213
Length	0.015	0.904	− 0.023	58.000	0.982	− 1.813	78.634	− 211.237	207.610
			− 0.023	57.981	0.982	− 1.813	78.634	− 211.240	207.613
Volume	0.07	0.932	0.26	57.000	0.991	0.030	1.166	1.166	3.136
			0.26	56.850	0.991	0.030	1.166	1.166	3.137

Table 1 showed the statistics of average value, standard deviation and standard error of the data measured by *Labview* and *WinRHIZO 2016*. The independent sample T test included the levin test results of the variance homogeneity. From the variance homogeneity levin test results of root surface area, average root diameter, root length and root volume, it could be seen that the data variance obtained from the two measurement methods was equal; assuming the T test results with equal variance, i.e. All the *P* values were greater than the significance level of 0.01, it could be judged that there was no significant difference in root surface area, average root diameter, root length and root volume measured by the two methods.

### Error analysis

In this study, *WinRHIZO 2016* (Canada) was used as the test standard to calculate the standard deviation, absolute error and relative error of root surface area, average diameter, length and volume measured by the software. The calculation formulas were as following:

The standard deviation (*S*) is

1 $$S = \sqrt {\frac{{\sum\nolimits_{i = 1}^{n} {\left( {x_{i} - \overline{x} } \right)^{2} } }}{n - 1}} .$$

The absolute error (*∆*) is

2 $$\Delta = \sum\limits_{i = 1}^{n} {x_{i} - \sum\limits_{i = 1}^{n} {y_{i} } } .$$

The relative error (*δ*) is

3 $$\delta = \frac{{\sum\nolimits_{i = 1}^{n} {x_{i} } - \sum\nolimits_{i = 1}^{n} {y_{i} } }}{{\sum\nolimits_{i = 1}^{n} {y_{i} } }} \times 100\% ,$$

*n* is the total number of samples, *x*_*i*_ is the parameters value of the *i*th sample measured by *Labview*, ${y}_{i}$ is the parameters value of the *i*th sample measured by *WinRHIZO 2016*.

After calculation, the results were showed as follows (see Table 2):

**Table 2:** Error analysis table

	Standard deviation *S*		Absolute standard error *∆*	Relative error *δ* %
	Labview	WinRHIZO 2016		
Surface area	146.520	148.300	83.355	0.480
Diameter	0.309	0.309	0.031	0.083
Length	307.310	301.750	54.390	0.080
Volume	4.440	4.440	0.389	0.110

The relative error was the value obtained by multiplying the ratio of the absolute error caused by the measurement to the measured [conventional] true value with 100%. In general, the relative error could better reflect the degree of confidence in the measurement. From the results of Table 2, the relative errors were 0.48% for root surface area, 0.083% for average root diameter, 0.08% for root length and 0.11% for system volume.

### Instrument calibration

**Fig. 1:**
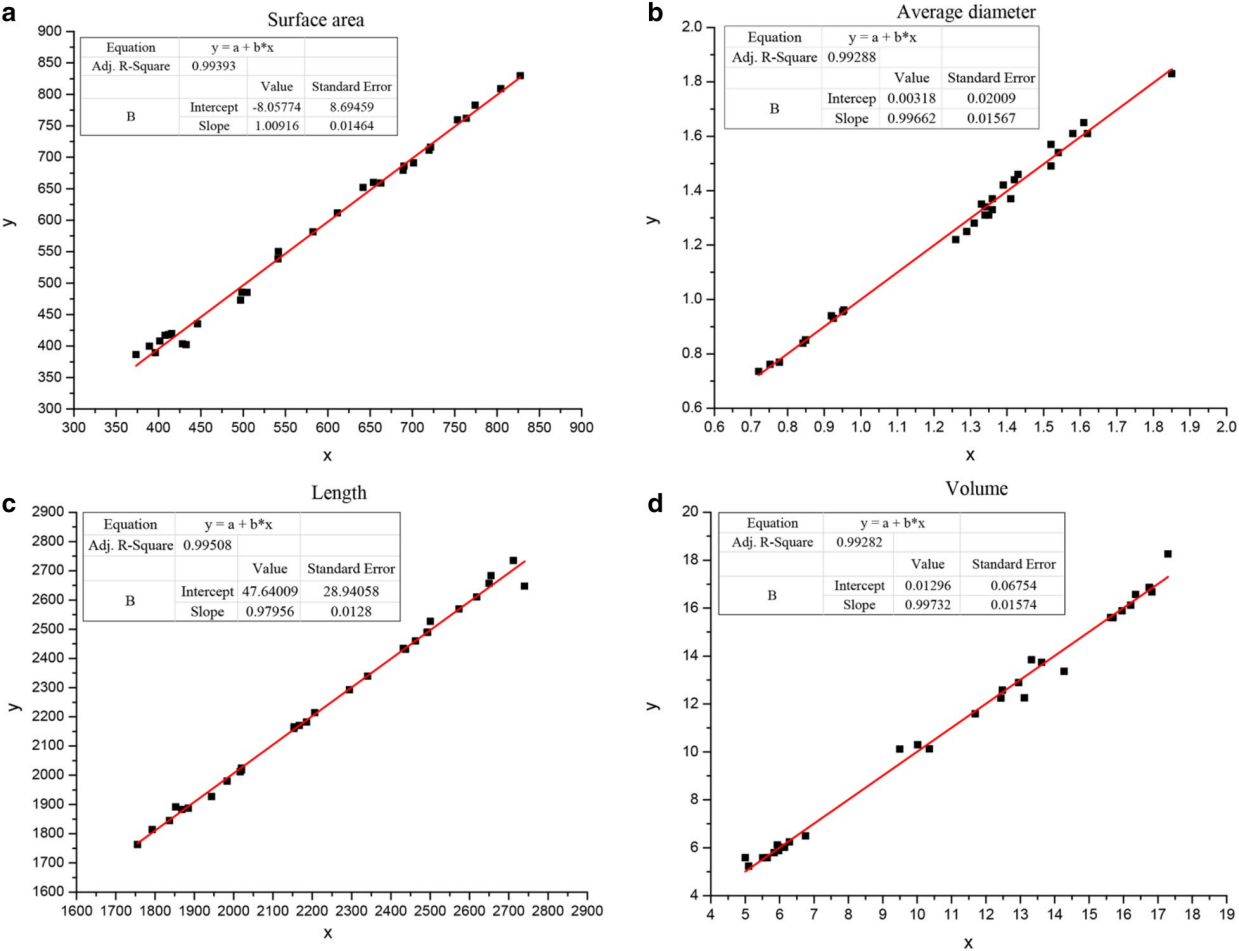
Comparison of the two measurement methods. x, *Labview* measurement value; y, *WinRHIZO 2016* measurement value; **a** root surface area analysis chart; **b** average root diameter analysis chart; **c** root length chart; **d** root volume analysis chart

*Labview* and *WinRHIZO 2016* were set under the same conditions to correct the measurement. The measurement deviation of the two measurement methods under the same conditions was calculated by the measurement of 30 samples, and the measurement deviation between the systems was corrected (see Fig. 1).

The measurement results showed that the two measurement methods had a stable linear relationship, and the *Labview* data was corrected based on the *WinRHIZO 2016* measurement value. The gain, offset and determination coefficients of each parameters were calculated as follows (see Table 3):

**Table 3:** Calibration data

Measurement parameters	Gain	Offset	Determination coefficient
Surface area	1.0092	− 8.0577	0.99393
Diameter	0.9966	0.0032	0.99288
Length	0.9796	47.6400	0.99508
Volume	0.9973	0.0432	0.99282

The gain, offset and determination coefficients of each parameter were calculated

## Discussion

The growth of plants is mainly derived from the absorption of and transportation of water and nutrients by roots. Root growth and its distribution not only affect the absorption of water and nutrients of root, but also directly affect the growth of fruit trees and the quality and yield of apples. At present, machine vision is mainly concentrated in the upper part of the study of plants, such as the measurement of sunflower flower size by image processing [3], the classification of potato quality by machine vision and three-dimensional image [4], the estimation of apple fruit size by 3D technology [5], etc. The physical measurement of root morphological parameters mainly relies on the root scanners currently available in the market. It requires a fixed hardware scanner and software [7]. Which is not only used to research the relationship between roots and soils [8], plant roots and water-saving irrigation [9], plant nutrition and water absorption [10], but also research the effects of different soils on main and lateral roots. This study developed and tested an image processing system to measure the morphological parameters of apple seedling roots (surface area, average diameter, length, and volume) accurately. The selected background plate facilitated the collection of seedling root images. The morphological parameters algorithm performed measurement according to the user input background plate size and produced measurement results. The datum got from the designed system and the pre-existing system *WinRHIZO 2016* was compared. The two-group datum was analyzed on the basis of the Independent-Samples T test. The result showed that the maximum *t* value was 0.073 and the minimum *t* value was 0.013, and all the significant (Double tail) values were greater than 0.01 (*P* > 0.01), indicating that there was no difference. The correlation between the two is extremely high. In the error analysis, the maximum relative error was 0.48% and the minimum was 0.08%, indicating little difference. In the result analysis, the measurement results also differed for different parameters. Compared with *WinRHIZO 2016* measurement results, measurement results of root surface area, average root diameter, root length and root volume *R*^2^ in this analysis system were 0.994, 0.993, 0.995 and 0.993, indicating that the difference was not significant. The difference between the same parameters was mainly embodied in root systems of different types, while the difference between the same types of root systems was small, and the difference between the measured values of the same root system also seemed tiny. This analysis software system designed could be used to measure the morphological parameters of apple root system.

## Conclusion

In this study, a set of measurement system for root morphology of apple seedlings was designed. This system employed the *Labview* software platform adjusted by statistical methods to accurately measure the surface area, average diameter, length and volume of apple root systems using machine vision technology. It was not only easy to operate, but also inexpensive, which had reduced the cost by 80–90% compared with the conventional scanner. The system could detect the main morphological parameters of apple seedlings roots (including the root size) and provided quality images. In addition, this research provided more intuitive and convenient method for the study of morphological parameters of apple seedling roots. The associated hardware equipment was simple to operate and requires no regular maintenance. The data resulted from this system supported researchers to effectively determine the growth status of apple seedling roots (normal growth roots were better than diseased roots) and the soil physical and chemical properties. This was of great significance to the traditional cultivation and management of apple seedlings.

## System design

**Fig. 2:**
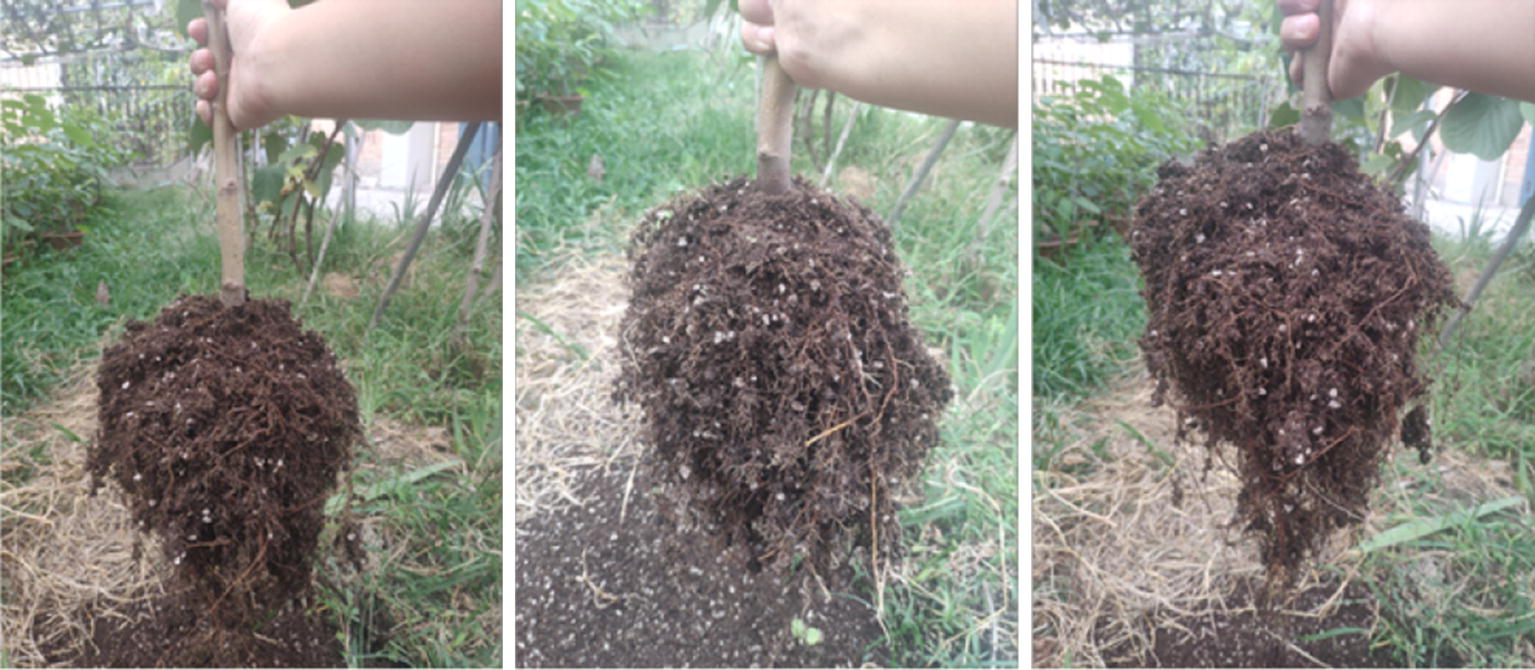
Seedling root collection

In this study, 2-year-old apple seedlings were used as research objects. Two-year-old B9 layering roots (asexually propagated seedlings, with small and dense roots), 2-year-old SH40 roots (tissue culture rooting seedlings) and 2-year-old B9 seedling roots (seedling plant) were selected. The seedlings were obtained from Li County of Baoding City, Hebei Province, China (38° 33′ 10.5ʺ N 115° 29′ 57.4ʺ E) (see Fig. 2). The meaningful part was extracted by background plate selection, image acquisition and image preprocessing. The surface area, average diameter, length and volume of the root system were obtained by the root characteristic parameters algorithm.

**Fig. 3:**
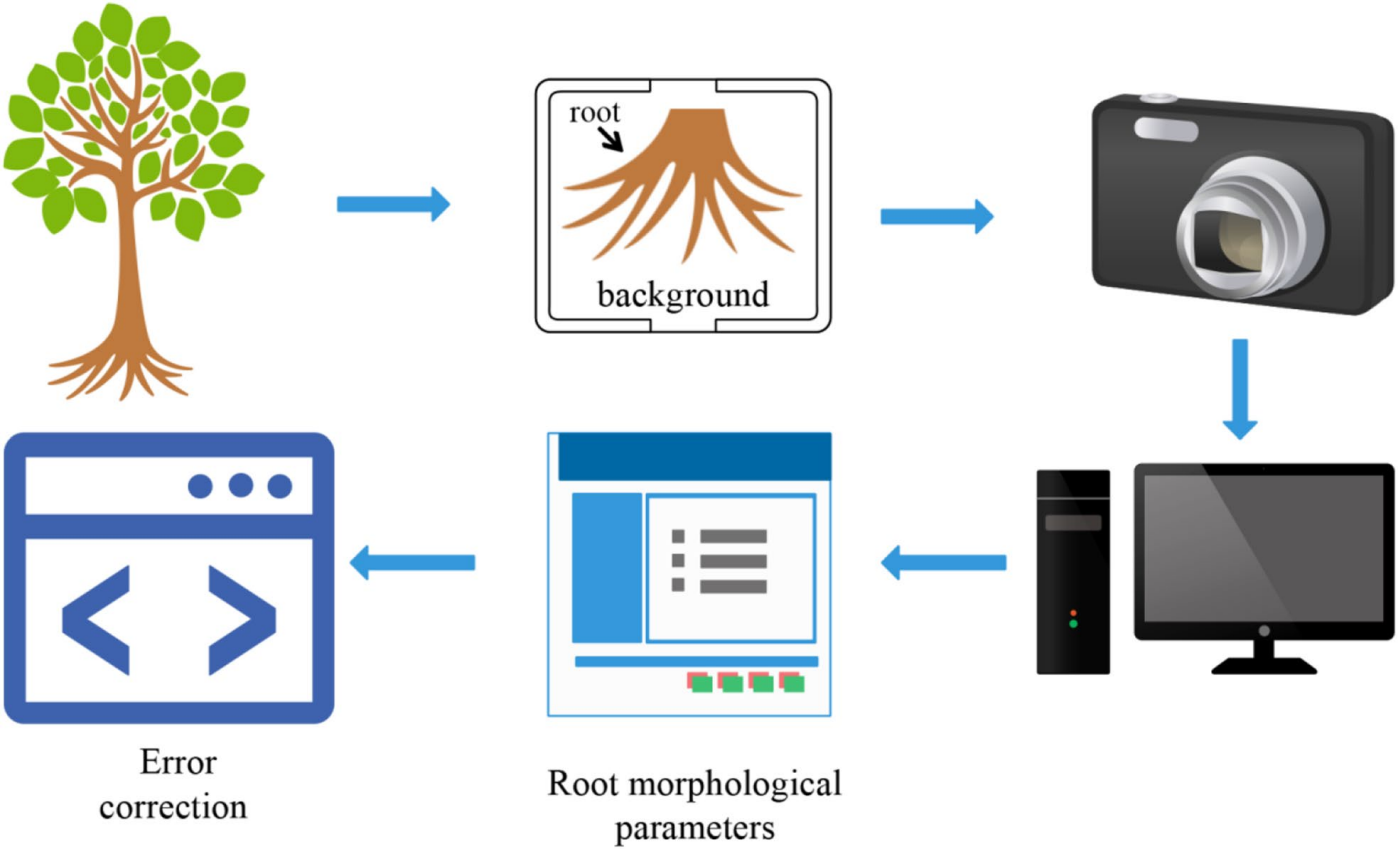
Schematic diagram of the system principle

The schematic diagram of the system principle was as follows (see Fig. 3 and Additional file 1: Figure S1).

### Hardware design

#### Background plate selection

Obtaining digital images from seedlings was challenging because the resulting images can suffer from image distortion, shadowing and insufficient contrast. In order to fully and clearly display the root image and facilitate segmentation, the background with significant contrast from the root color should be selected to enhance contrast of the acquired image [3]. At the same time, the selected background board should be larger than the seedling root and the actual area should be known. In this study, because the apple roots were darker in color and root types were different, white background board of 297 × 420 mm and 610 × 420 mm were selected [4].

#### Image collector selection

Pixel resolution referred to the minimum number of pixels required to display a complete object to be measured and to display an image of useful information points on the object being measured. The pixel resolution could be determined by the smallest eigenvalue of the detected object [11]. According to the following formula:

4 $$Pixel \, resolution = \left( {\frac{the \, maximum \, length \, of \, the \, detected \, object}{{the \, minimum \, eigenvalue \, of \, the \, object}}} \right) \times 2.$$

Assuming that the maximum length of the root system was 300 mm and the minimum eigenvalue was 1 mm, the minimum pixel resolution required to calculate according to the above formula was 600.

In order to make the image clear without shadow, the selected data acquisition device was Canon SX620, with 20.2 million pixels [12], 25 mm wide angle, 25-fold optical zoom, highest resolution of 5184 × 3888, ISO80-3200 and optical image stabilization. In *RGB* color mode, the image collector should directly face the center of the background plate during the shooting process, and the background plate should be included in it for collection.

### Software design

**Fig. 4:**
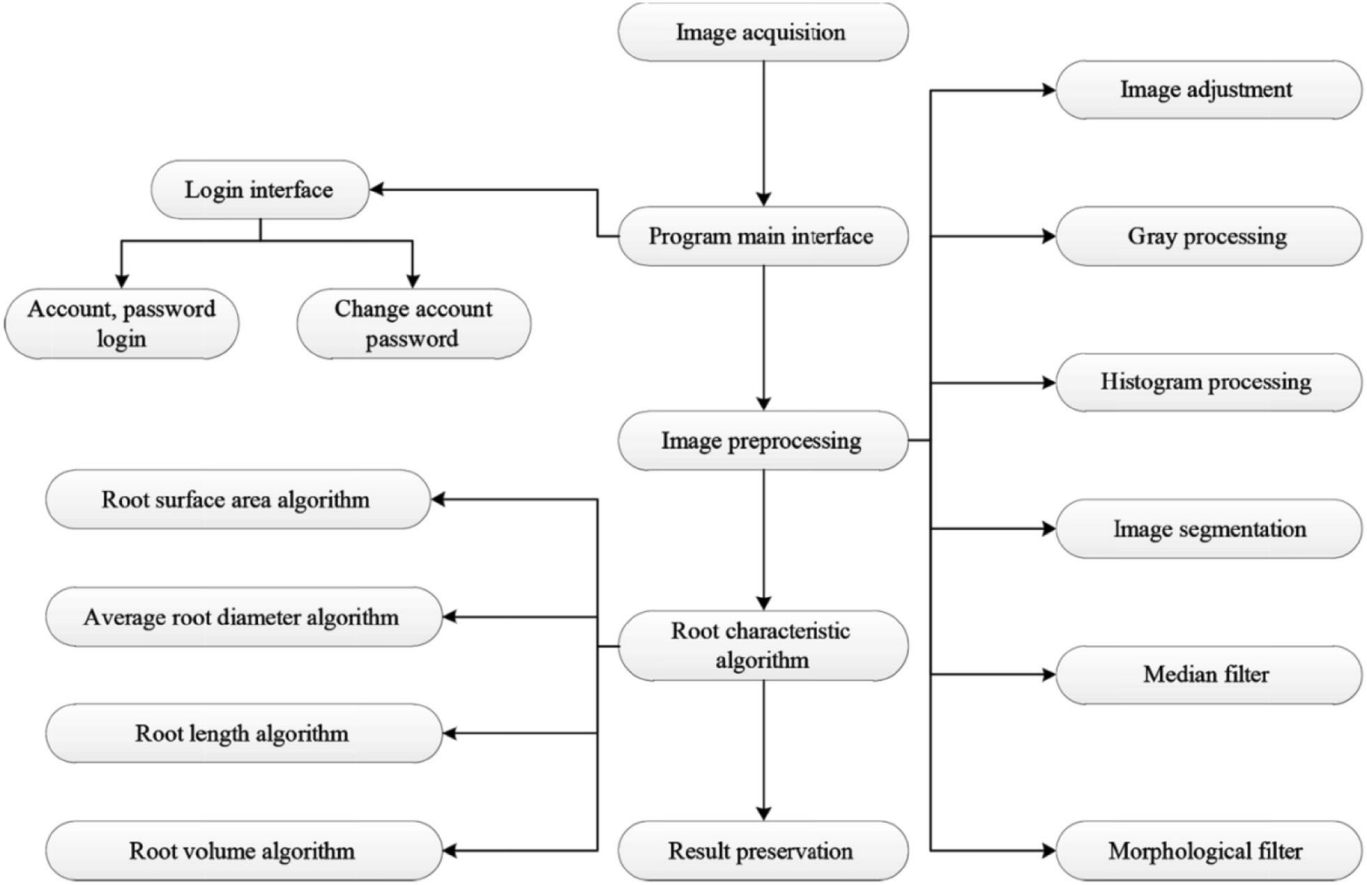
System flow chart

The system mainly included a login module, a database module [13] and an image preprocessing module, a data analysis processing module and a system result output module. The image processing module mainly included image cropping, gray processing, image histogram, image segmentation, median filter and morphological filter (see Fig. 4).

#### Image cropping

In the image acquisition process, due to the different acquisition positions, parts outside the background plate may appear in the captured image. In order to ensure data accuracy, the image needed to be cropped while retaining the background plate and corresponding pixel values [3].

#### Gray processing

The image acquired by the image collector was a color image, and the model usually oriented to the hardware was *RGB* model [14]. *RGB* was to adjust color from the principle of optics, which did not reflect the morphological characteristics of the image, so the color image should be converted into an 8-byte grayscale image for processing [15]. A grayscale image was a component of a color image, which was of 8 bytes [16], and each pixel was indicated by 0–255. To make the images and results more accurate, this study adopted 8-byte grayscale images. According to the following formula:

5 $$Gray = R \times 0.299 + G \times 0.587 + B \times 0.114.$$

*Gray* is the grayscale image; *R* is the red component map in the *RGB* image; *G* is the green component map in the *RGB* image; *B* is the blue component map in the *RGB* image.

It had been experimentally proved that the *R* component map and the *G* component map could not clearly distinguish the background from the root system, so *B* component was extracted to complete the conversion from color image to grayscale image.

#### Histogram processing

The histogram [17] could be used not only for image enhancement, but also for image segmentation. If there was a distinct bimodal shape in the gray histogram, the gray value corresponding to the valley between the two peaks could be selected as the threshold [11]. However, this method was not suitable for the case where the bimodal difference in the histogram was great or the valley between the double peaks was relatively broad and flat, as well as the case of unimodal histogram. If the above two histograms appeared, the iterative threshold method should be selected to obtain the optimal threshold. According to the following formula:

6 $$g\left( {x,y} \right) = \left\{ \begin{array}{*{20}l} 1\quad f\left( {x,y} \right) > t \hfill \\ 0\quad other \hfill \\ \end{array}, \right.$$

where *t* is the threshold value.

#### Image segmentation

The main purpose of image segmentation was to separate the desired target image from the background region so that the target image could be extracted [18, 19]. Due to the root system and its irregularity, neither the boundary detection method nor the region extraction method could effectively separate the image from the background. Therefore, threshold segmentation method was selected in this study [20–22].

In order to solve the case that some images could not show obvious double peaks in the histogram and reduce the error segmentation probability, the optimal threshold was obtained by the algorithm of iterative threshold [23]. According to the histogram, the gray value range was selected as the initial threshold *T*_*0*_. Assuming that there were a total of *L* gray values, iteration was performed according to the following formula:

7 $$T_{i + 1} = \frac{1}{2}\left\{ {\frac{{\sum\nolimits_{k = 0}^{{T_{i} }} {h_{k} \times k} }}{{\sum\nolimits_{k = 0}^{{T_{i} }} {h_{k} } }} + \frac{{\sum\nolimits_{{k = T_{i} }}^{L - 1} {h_{k} \times k} }}{{\sum\nolimits_{{k = T_{i + 1} }}^{L - 1} {h_{k} } }}} \right\},$$

where *L* is the number of gray levels, and *T*_*i*_ is the threshold after iteration; *h*_*k*_ is the number of pixels whose gray value is *k*. The iteration termination condition is $T_{i + 1} = T_{i}$. When the iteration ends, *T*_*i*_ is taken as the optimal segmentation threshold *T*.

#### Median filter

The captured image was subject to the influence of light source, jitter, external signal interference, etc., often with impulse noise, resulting in blurred image, loss of detail and resolution degradation, affecting the analysis results. Therefore, it was necessary to filter the captured photos [15, 24]. The median filter method was a common method to remove impulse noise [11], which could eliminate isolated noise points, provide excellent noise reduction performance, and effectively protect image edge features. Two-dimensional median filter output is

8 $$g\left( {x,y} \right) = med\left\{ {f\left( {x - k,y - 1} \right),\left( {k,1 \in w} \right)} \right\},$$

where $f\left( {x,y} \right),g\left( {x,y} \right)$ are respectively original image and processed image; *w* is a two-dimensional template.

#### Morphological filter

The language of mathematical morphology was a set theory. As a tool to extract image components useful for expressing and describing regions from images, such as boundaries, skeletons, etc., shape expansion and corrosion were the basis of mathematical morphological processing. Expansion was mainly to fill in some small cavities in the pixels appearing in the roots that were not captured during the threshold processing. The expression was to assume *A* and *B* were a set in *Z*^*2*^, and *A* expansion by *B* was defined as:

9 $$A \oplus B = \left\{ {z\left| {\left[ {\left( {\widehat{B}} \right)_{z} \cap A} \right]} \right. \subseteq A} \right\}.$$

Corrosion was a process of eliminating boundary points and shrinking the boundaries to the interior, which could be used to eliminate tiny and meaningless objects [25]. The main purpose was to eliminate some very small debris pixels that may appear during threshold processing. The expansion expression was to assume both *A* and *B* were a set in *Z*, which were expressed as *A*Θ*B* and defined as:

10 $$A\Theta B = \left\{ {z\left| {\left( B \right)_{z} \subseteq A} \right.} \right\}.$$

This formula showed that the use of *B* to etch *A* was to let the set of points *z* in *B* contained in A be translated by *z*.

Expansion and corrosion were mutually dual for the set complement and reflection operations, that was:

11 $$\left( {AB} \right)^{c} = A^{c} \oplus B^{c} .$$

The open operation was the process of first corrosion and then expansion [26]. Using the structural element *B* to open the set *A*, the expression was:

12 $$A \circ B = \left( {A\Theta B} \right) \oplus B.$$

The open operation could be used to eliminate small objects, separate objects at slender points, and not significantly change the area while smoothing the boundaries of larger objects.

The closed operation was the process of first expansion and then corrosion [27], and the expression was:

13 $$A \bullet B = \left( {A \oplus B} \right)\Theta B.$$

Closed operations were used to fill small cavities in objects, connect adjacent objects and smooth the boundaries without significantly changing the area.

**Fig. 5:**
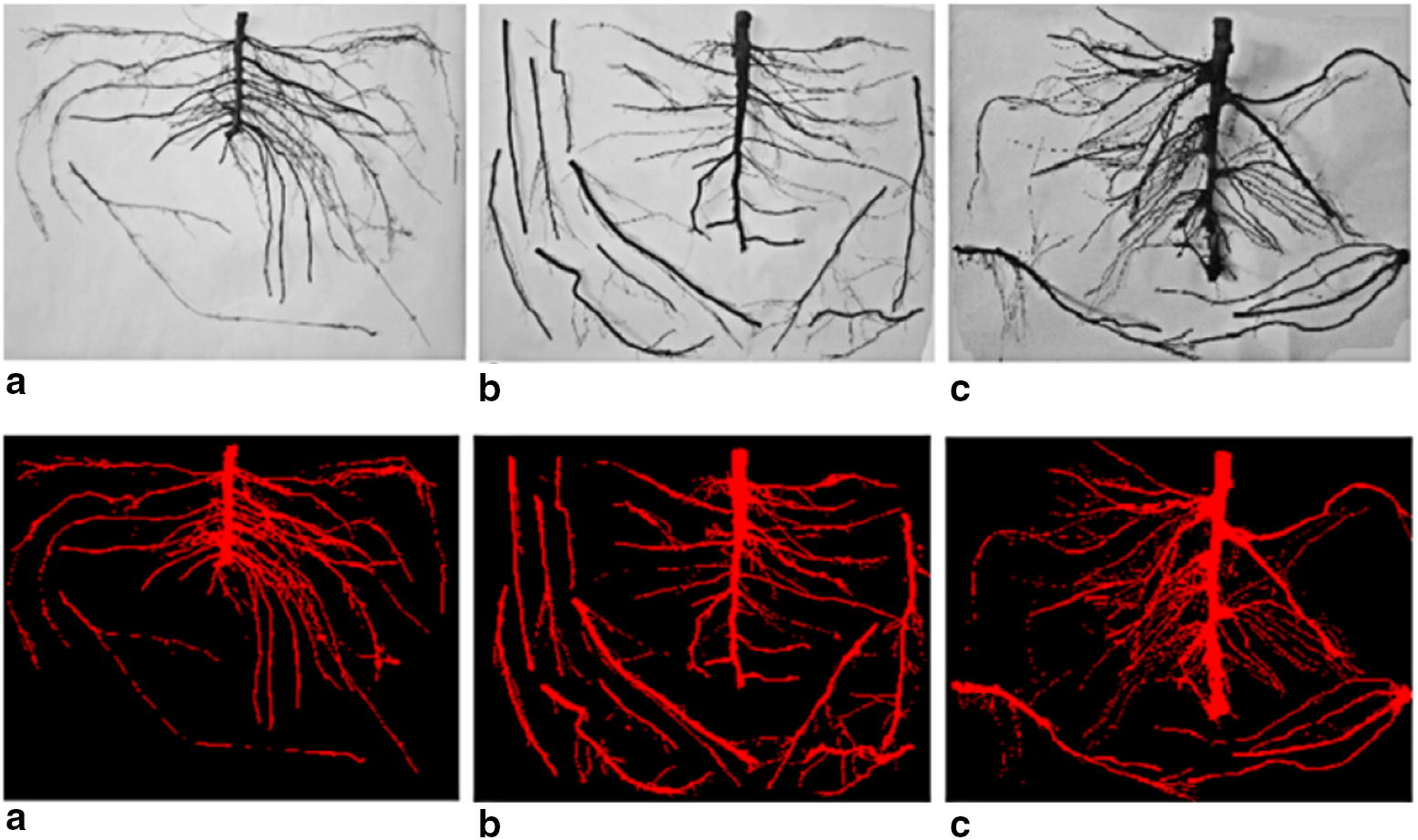
Preprocessing image. **a** comparison of B9 layering root image before and after processing; **b** comparison of SH40 root image before and after processing; **c** comparison of B9 seedling root system before and after processing

The system mainly applied expansion corrosion, open and closed operations in binarized image morphology processing to fill small cavities and eliminate fine debris pixels (see Fig. 5).

#### Calculation of root surface area

The acquired root image was a planar graph, which could be used as a vertical section of the root system, which was a segmented image obtained in image processing. It could also be regarded as a projected area. According to the basic theory of plant nutrition [28], plant roots were uniform in longitudinal growth, so each cross-section of the root system could be considered as a circle. According to the established cylindrical approximation model [29], the root system could be regarded as a three-dimensional figure formed by stacking many small cylindrical micro-elements. The overall characteristic parameters of the root system could be calculated based on the model.

Assuming that the number of pixels with pixel values greater than 1 in the segmented image was *N*_*a*_; the number of pixels was *n*; the projected area of the root system could be expressed as:

14 $$S_{vertical} = n \times N_{a} .$$

The root system could be seen as a stack of *m*-segment small cylindrical micro-elements (*m* is a finite number). The surface area was the cumulative sum of the side areas of the *m*-segment cylinder, i.e.:

15 $$S_{lateral} = \pi \times S_{vertical} \times m.$$

#### Calculation of average root diameter

The image was a planar graph and had been previously segmented. In order to calculate the radius of the root system, the circle detection was used by setting the radius to 0–100 pixels, and then the radius of all the roots could be captured to find the average diameter of the root system. Assuming that the number of detected circles was *n* and the radius was *m*, the average diameter *d* was:

16 $$D = 2r = \frac{{\sum\nolimits_{i = 1}^{n} m }}{m} \times 2.$$

#### Root length calculation

In order to facilitate the calculation of root length, the image could be refined to approximate the skeleton line of the object [30]. The refined image forms the skeleton of the image, and the refined image width was one pixel, while the length and the original image were kept unchanged. The length of the entire root system could be obtained by counting the number of pixels and multiplying it with the distance between the pixels. In this experiment, the chain length statistical method was used to obtain the length information of the root system.

The collected roots were irregular images which could be calculated in 8-connected mode. In the 8-connected mode, the distance between each pixel and its neighboring pixels was not equal. Assuming that any pixel had a distance of 1 from the adjacent pixels in the vertical direction and the horizontal direction, the distance of the pixel in the oblique line direction was $\sqrt 2$.

In this study, direction code was used to distinguish two different spacings: if the adjacent two pixels were even, the distance was 1; if the adjacent two pixels were odd, then the distance was $\sqrt 2$. If *N*_*1*_ and *N*_*2*_ were separately calculated in different ways, and *C* was the calibration coefficient of the root length, then the length of the root system was:

17 $$L = C\left( {N_{1} + N_{2} \times \sqrt 2 } \right).$$

#### Calculation of root volume

The average diameter and length of the root system were calculated in “Calculation of average root diameter” and “Root length calculation”, respectively, and the root system was considered as a solid figure stacked by small cylinders as mentioned in “Calculation of average root diameter”. The volume *V* of the root system was:

18 $$V = \sum\limits_{i = 1}^{n} m /m \times C\left( {N1 + N2\sqrt 2 } \right).$$

**Fig. 6:**
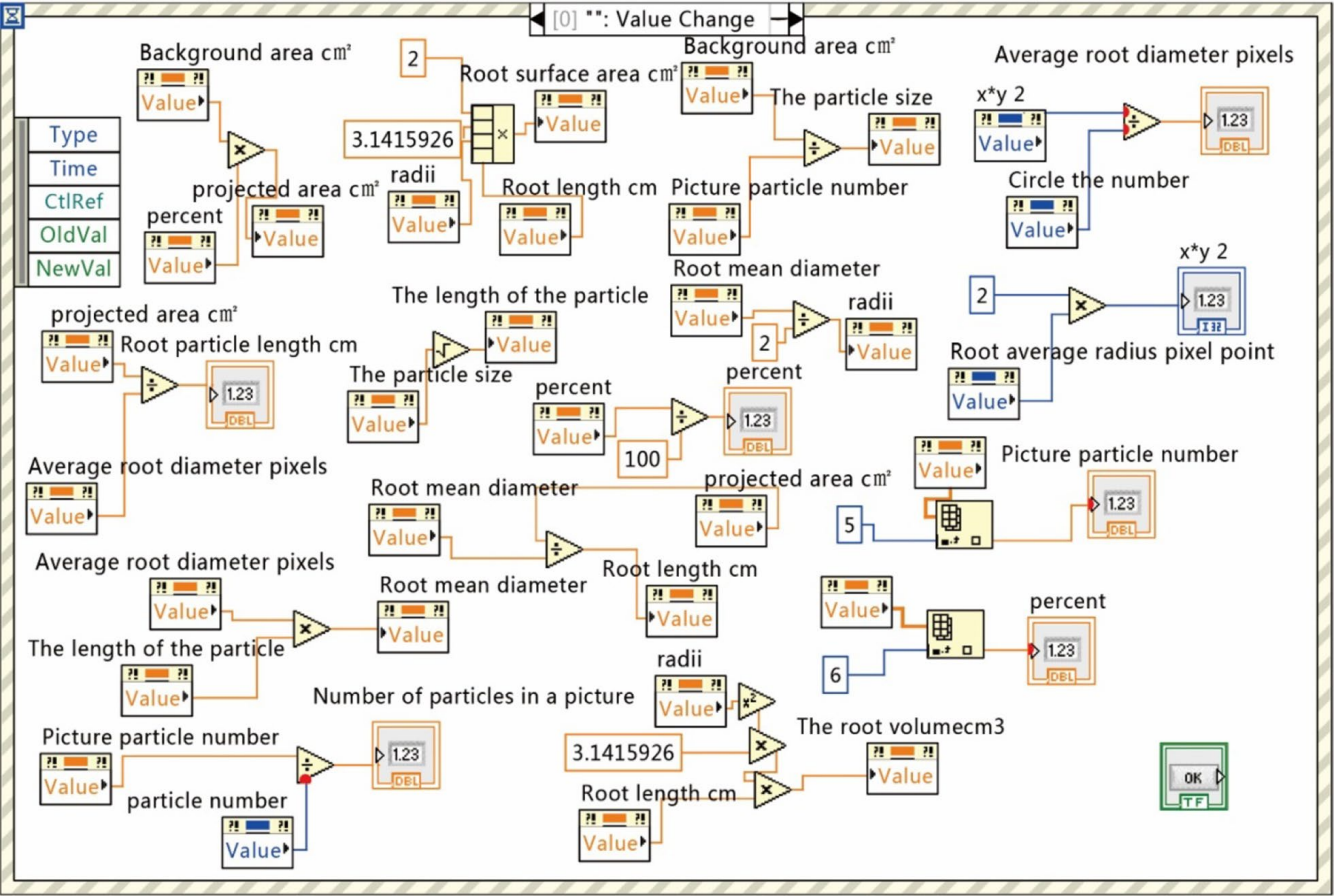
Program code

Part of the program code is shown in Fig. 6.

## Supplementary information

**Additional file 1.** Framework figure of the research.

## Data Availability

The datasets generated during and/ or analyzed during the current study are not publicly available due to the Metadata was computer codeData but are available from the corresponding author on reasonable request.
