# Supplementary figures and images for: Study on measurement method for apple root morphological parameters based on Labview
Source: Plant Methods. 2019 Dec 11;15:149. doi: 10.1186/s13007-019-0535-4 (PMC6905017; doi:10.1186/s13007-019-0535-4)

# Additional File

Additional file Figure S1:


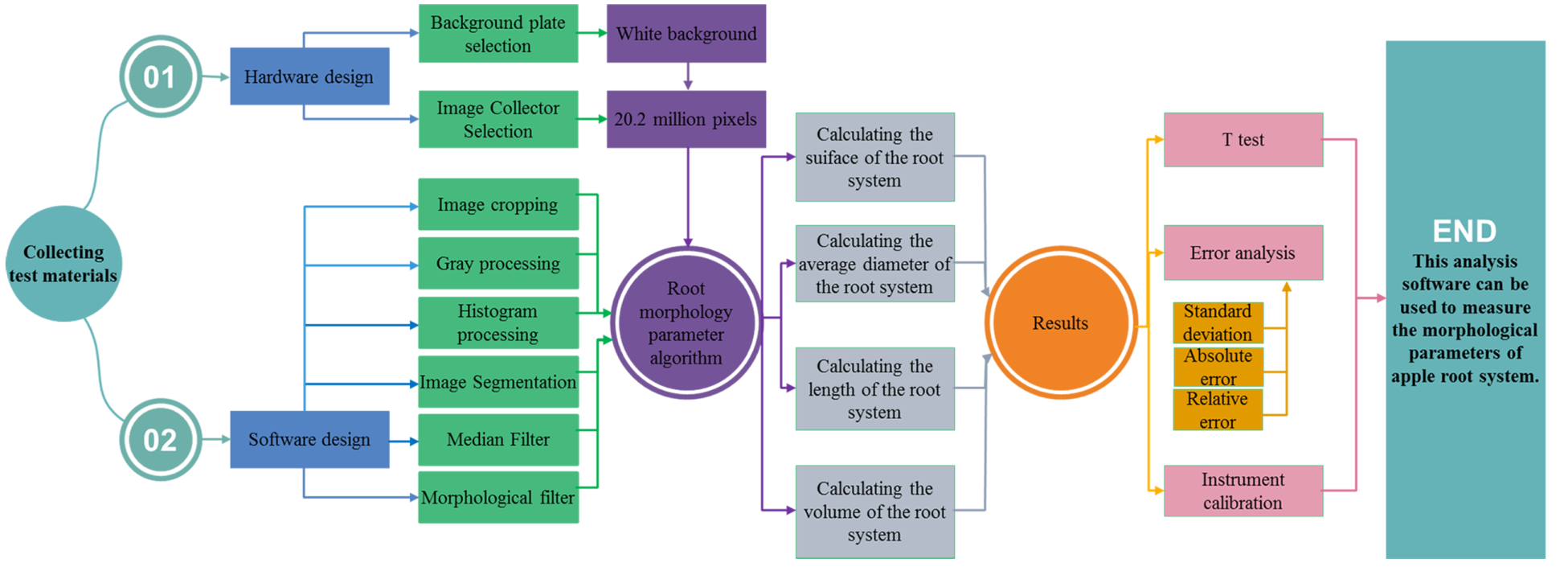

Supplement: Supplementary file 1 — Additional file 1. Framework figure of the research. [file 13007_2019_535_MOESM1_ESM.docx]
